# Special Issue “Innovative Techniques and Approaches in the Control and Prevention of Rabies Virus”

**DOI:** 10.3390/v14050845

**Published:** 2022-04-19

**Authors:** Amy T. Gilbert, Ryan M. Wallace, Charles E. Rupprecht

**Affiliations:** 1 United States Department of Agriculture, Animal and Plant Health Inspection Service, Wildlife Services, National Wildlife Research Center, Fort Collins, CO 80521, USA; 2 Poxvirus and Rabies Branch, Division of High Consequence Pathogens and Pathology, United States Centers for Disease Control and Prevention, Atlanta, GA 30333, USA; 3 LYSSA LLC, Lawrenceville, GA 30044, USA

Rabies is an ancient lethal scourge that has plagued humankind for centuries. Globally, 60,000 human deaths are estimated to occur each year from rabies virus (RABV) transmission in domestic dogs, mostly affecting children. While rabies is recognized as a neglected disease, there is cause for optimism in the context of growing global recognition, collaboration and commitment to advance a tripartite agenda to eliminate human deaths transmitted from rabid dogs by 2030, also known as “Zero By Thirty” (ZBT). Nevertheless, the ZBT goal must also confront competing challenge(s) of tracking and mitigating human morbidity and mortality during a global pandemic caused by a viral zoonosis with likely origins from one or more wildlife reservoirs. In this context, the concept of One Health has never been more relevant and symbolic as demonstrated with prevention, control and elimination to end human rabies deaths through the mass vaccination of domestic and wild animal reservoir populations.

With an anticipated global reduction in human rabies burden through deployment of integrated vaccination programs and approaches principally targeting control of RABV in both owned and free-roaming dog populations [1], surveillance efforts may shift to include at-risk wildlife populations (e.g., meso-carnivores and bats). Tracking RABV infection status and epizootiology in wildlife reservoir populations has implications for public and animal health [2] and the deployment of field-friendly, sensitive and specific detection and characterization systems will advance insights on RABV pathogenesis and ecology in wildlife reservoir populations to assess the risks of spillover transmission to humans, domesticated animals, and threatened or endangered wildlife.

Our Special Issue of *Viruses*, *Innovative Techniques and Approaches in the Control and Prevention of Rabies Virus*, presents a diverse collection of peer-reviewed articles that advance knowledge and the practical application of technical methods and tools towards the prevention and control of rabies in humans, dogs, and wildlife. We learn about differences in expression patterns of 120 genes following experimental RABV infection and likely contributing to variable pathogenicity of dog versus vampire bat RABV [3]. Another report evaluates the binding and neutralizing activity of two mouse-derived monoclonal antibodies (15–13 and 12–22) against RABV in vitro, in support of research and development on alternatives to human rabies immune globulin for post-exposure prophylaxis [4]. Another set of articles highlight the context and principles guiding the interpretation and significance of RABV serology in humans and animals [5], particularly in comparing evidence of immunogenicity between intradermal versus intramuscular vaccination regimens for humans [6].

In looking to the field, we learn of recent advances in parenteral vaccine delivery strategies and programs for owned dogs in Tanzania [7], as well as proof-of-concept field trials of oral rabies vaccination focused upon free-roaming dogs in Thailand [8]. We learn about practical applications of oral vaccination biomarkers in dogs [9] and sample collection and diagnostic methods for monitoring owned and free-roaming dog populations following vaccination campaigns utilizing parenteral and oral delivery methods [10,11]. This issue also expands on surveillance, control, and oral vaccination product and strategy evaluations for use with challenging wildlife targets like mongooses [12,13], skunks [14] and bats [15,16], including hematophagous species.

We thank all of the contributing authors for evidence-based research to advance laboratory-based surveillance, prevention and control of a highly lethal and neglected viral zoonosis through application of modern management principles to domestic and wild animal reservoirs in a One Health context.
